# SISH/CISH or qPCR as alternative techniques to FISH for determination of *HER2* amplification status on breast tumors core needle biopsies: a multicenter experience based on 840 cases

**DOI:** 10.1186/1471-2407-13-351

**Published:** 2013-07-22

**Authors:** Jocelyne Jacquemier, Frédérique Spyratos, Benjamin Esterni, Marie-Joëlle Mozziconacci, Martine Antoine, Laurent Arnould, Sarab Lizard, Philippe Bertheau, Jacqueline Lehmann-Che, Cécile Blanc Fournier, Sophie Krieger, Frédéric Bibeau, Pierre-Jean Lamy, Marie Pierre Chenard, Michèle Legrain, Jean-Marc Guinebretière, Delphine Loussouarn, Gaëtan MacGrogan, Isabelle Hostein, Marie Christine Mathieu, Ludovic Lacroix, Alexander Valent, Yves Marie Robin, Françoise Revillion, Magali Lacroix Triki, Aline Seaume, Anne Vincent Salomon, Patricia de Cremoux, Geneviève Portefaix, Luc Xerri, Sophie Vacher, Ivan Bièche, Frédérique Penault-Llorca

**Affiliations:** 1 Institut Paoli Calmettes, biopathology department, 232 Bd Ste Marguerite, 13009, Marseille, France; 2 Institut Curie - Hôpital René Huguenin, 35 rue Dailly, 92210, Saint Cloud, France; 3 Hôpital Tenon, 4 rue de la Chine, 75970, Paris, France; 4 Centre Georges-François Leclerc, 1 rue du Professeur Marion, 21079, Dijon, France; 5 Hôpital St-Louis, 1 rue Claude Vellefaux, 75475, Paris, France; 6 Centre François Baclesse, 3 avenue du Général Harris, 14076, Caen, France; 7 Centre Val D’Aurelle, 31 rue de la Croix Verte, Parc Euromédecine, 34298, Montpellier, France; 8 Hopital Haute Pierre, Avenue Molière, 67098, Strasbourg, France; 9 Hopital Laennec, Bd Jean Monod, 44800, St Herblain, France; 10 Institut Bergonié, 229 cours de l’Argone, 33076, Bordeaux, France; 11 Institut Gustave Roussy, 39 rue Camille Desmoulins, 94805, Villejuif, France; 12 Centre Oscar Lambret, 3 rue Frédéric Combemale, 59020, Lille, France; 13 Institut Claudius Regaud, 20-24 rue du Pont Saint-Pierre, 31052, Toulouse, France; 14 Institut Curie Paris, 26 rue d’Ulm, 75248, Paris, France; 15 present address: Hôpital St-Louis, 1 rue Claude Vellefaux, 75475, Paris, France; 16 Centre Jean Perrin, EA 4677 Clermont-Ferrand, 58 rue Montalembert, 63011, Clermont-Ferrand, France

**Keywords:** HER2, FISH, SISH, CISH, qPCR, Multicenter analysis

## Abstract

**Background:**

Until now, FISH has been the gold standard technique to identify *HER2* amplification status in ambiguous cases of breast cancer. Alternative techniques have been developed to increase the capacities of investigating *HER2* amplification status. The aims of this multicenter study in a large series of breast cancer patients were to prospectively compare the level of performance of CISH, SISH, and qPCR alternative techniques on paraffin-embedded core biopsies with “gold standard FISH” for evaluation of *HER2* amplification status.

**Methods:**

This study was performed on 840 cases scored by immunohistochemistry (IHC): 0=317 (38%), 1+=183 (22%), 2+=109 (13%), 3+=231 (27%). Each of the 15 French centers participating in the study analyzed 56 breast carcinoma cases diagnosed on fixed paraffin-embedded core biopsies. *HER2* amplification status was determined by commercially available FISH used as the reference technique with determination of the HER2/CEN17 ratio or *HER2* copy number status. The alternative techniques performed on the same cases were commercially available SISH or CISH and a common qPCR method especially designed for the study including a set of 10 primer pairs: 2 for *HER2* (exons 8 and 26), 5 to evaluate chromosome 17 polysomy *TAOK1*, *UTP6*, *MRM1*, *MKS1*, *SSTR2* and 3 for diploidy control *TSN*, *LAP3* and *ADAMTS16*.

**Results:**

The concordance between IHC and FISH was 96% to 95% based on the HER2/CEN17 ratio (n=766) or *HER2* copy number (n=840), respectively. The concordance of the alternative techniques with FISH was excellent: 97% and 98% for SISH (498 and 587 cases), 98% and 75% for CISH (108 and 204 cases) and 95% and 93% (699 and 773 cases) for qPCR based on the HER2/CEN17 ratio or *HER2* copy number, respectively. Similarly, sensitivity ranged from 99% to 95% for SISH, 100% to 99% for CISH and 89% to 80% for qPCR. The concordance with FISH (ratio) in the 2+ cases was 89% for SISH, 100% for CISH and 93% for qPCR.

**Conclusion:**

These alternative techniques showed an excellent concordance with FISH in core biopsies allowing their use in routine clinical practice. This newly designed qPCR on paraffin-embedded core biopsies deserves special attention, as it is reliable, easy to perform and less expensive than ISH tests.

## Background

*HER2* overexpression occurs in 14% to 20% of early breast cancers. The poor prognosis initially described for these *HER2*-positive cases has been corrected by the development of a humanized monoclonal antibody, trastuzumab (HERCEPTIN®) that significantly improves the survival of patients with *HER2*-positive status, as demonstrated by numerous clinical trials [1,2]. Immunohistochemistry (IHC) using scoring tools such as the “Hercept scale” and, more recently, the ASCO/CAP scale, was the simplest way to identify positive cases likely to benefit from trastuzumab [3]. However, according to this scale, *HER2* amplification of 2+ cases had to be confirmed by Fluorescent in situ hybridization (FISH).

IHC is susceptible to interobserver variability and, as with any assay technique, required standardization and validation [3-10]. A very good correlation has been demonstrated between HER2 protein overexpression and *HER2* gene amplification [11]. Therapeutic response to trastuzumab was observed exclusively in patients harboring *HER2* gene amplification [12]. Some neoadjuvant studies suggested that the level of response was correlated with the level of gene amplification [13], while large-scale prospective adjuvant clinical trials failed to demonstrate this correlation [14]. *HER2* amplification status can be analyzed by FISH, which is a sensitive and specific method that identifies the number of copies of the *HER2* gene often in conjunction with the chromosome 17 centromere and is considered to be the “gold standard”. However, FISH is not readily available, requires very specific training [15], is time-consuming requiring the use of a fluorescent microscope and cytogenetic skills, and is also expensive. Chromogenic in situ hybridization (CISH) [16] and silver-enhanced in situ hybridization (SISH) [17-21] are new bright field techniques that have been more recently introduced for determination of *HER2* gene status. Quantitative real-time polymerase chain reaction (qPCR) is such a rapid, sensitive and quantitative alternative technique [22-29], requiring small amounts of tissue and which can be performed on paraffin-embedded samples. Moreover, it has a high throughput capacity.

The main objective of this multicenter study, based on large series of patients, was to prospectively compare the performance level of the CISH, SISH and qPCR alternative techniques on core biopsy specimens with the “gold standard FISH” for evaluation of *HER2* amplification status. The second objective was to conduct a medico-economic study, which is not reported in this paper. This study was conducted by 15 hospitals homogeneously distributed throughout France in the framework of a project entitled "Support Program for Costly Diagnostic and Therapeutic Innovations" supported by the French Institute of Cancer (INCa).

## Results

### Population characteristics

The mean age of the patients included in the study was 58.6 years; 92% of women had non-inflammatory breast cancer, and the mean clinical diameter of the lesion was 26.75 mm.

The study was confined to core biopsies performed before therapy: 89% of core biopsies were microbiopsies, including 81% of 14 G needle biopsies. The median value of tumor cellularity was 60% (5-100). The intraductal component represented a mean of 3.9%. Only 12% of core biopsies had a fixation time of less than 4 hours.

### Immunohistochemistry and FISH

IHC and FISH with *HER2* copy number were available for 840 breast cancer cases: 766 cases were analyzed by a double probe technique allowing calculation of both *HER2* copy number and HER2/CEN17 ratio. The remaining 74 cases were analyzed by a mono-probe technique only taking into account *HER2* copy number*.*

A strong correlation was observed between the ASCO/CAP score for IHC and the FISH level of amplification (Tables 1 and 2). On FISH, 223/766 (29%) cases had a ratio greater than 2.2 (Table 1) and 248/840 (29.5%) cases had an *HER2* copy number greater than 6 (Table 2).

**Table 1:** Distribution of the 766 cases analyzed by double probe FISH expressed as HER2/CEN17 ratio in 3 categories with respect to the CISH, SISH and QPCR alternative techniques

**Techniques**	**Class**	**Number of cases**	**FISH ratio <1.8 N=534 (%)**	**FISH ratio [1.8-2.2] N=9 (%)**	**FISH ratio >2.2 N=223 (%)**
IHC	0	287	282 (98.2)	3 (1)	2 (0.8)
	1+	172	166 (96.5)	3 (1.75)	3 (1.75)
	2+	95	77 (81)	2 (2.2)	16 (16.8)
	3+	212	9 (4.2)	1 (0.4)	202 (95.2)
SISH	<1.8	331	327 (98.8)	3 (0.9)	1 (0.3)
	[1.8-2.2]	11	7 (63.7)	3 (27.3)	1 (9)
	>2.2	156	10 (6.4)	1 (0.6)	145 (92.9)
	ND or NA	268	190	2	76
CISH	<1.8	75	74 (98.7)	1 (1.3)	0
	[1.8-2.2]	0	0	0	0
	>2.2	33	2 (6)	0	31 (94)
	ND or NA	658	458	8	192
qPCR	<1.8	492	471 (95.7)	5 (1)	16 (3.3)
	[1.8-2.2]	12	5 (41.7)	0	7 (58.3)
	>2.2	195	12 (6.2)	3 (1.5)	180 (92.3)
	ND or NA	67	46	1	20

ND: not done.

NA: not available.

**Table 2:** Distribution of the 840 cases analyzed by mono or double probe FISH expressed as HER2 copy number (cutoff set at 6 HER2 copies) with respect to the CISH, SISH and QPCR alternative techniques

**Techniques**	**Class**	**Number of cases**	**FISH Copies Number <6 N=592 (%)**	**FISH Copies Number >=6 N=248 (%)**
IHC	0	317	316 (99.6)	1 (0.4)
	1+	183	179 (97.8)	4 (2.2)
	2+	109	86 (78.9)	23 (21.1)
	3+	231	11 (4.8)	220 (95.2)
SISH	<6	417	409 (98)	8 (2)
	>=6	170	4 (2.4)	166 (97.6)
	ND or NA	243	179	74
CISH	<6	89	88 (98.8)	1 (0.2)
	>=6	115	49 (42.6)	66 (57.4)
	ND or NA	636	455	181
qPCR	<6	576	531 (92.2)	45 (7.8)
	>=6	197	12 (6)	185 (94)
	ND or NA	67	49	18

ND: not done.

NA: not available.

Among the 3+ IHC cases, 95.2% had an HER2/CEN17 ratio greater than 2.2, and 95.2% had an *HER2* copy number greater than 6. Among the 2+IHC cases, 16.8% had an HER2/CEN17 ratio greater than 2.2, 21.1% had an *HER2* copy number greater than 6 and 2.2% had a borderline HER2/CEN17 ratio (1.8-2.2). In the 1+ IHC category, 1.75% of cases had an HER2/CEN17 ratio greater than 2.2 and 1.75% of cases had a borderline ratio. Only 2.2% of the 1+ cases had an *HER2* copy number greater than 6. A similar correlation was obtained with the 0 IHC category, in which 0.8% of cases had an HER2/CEN17 ratio >2.2 and 0.4% of cases had an *HER2* copy number greater than 6.

As shown in Additional file 1: Table S3, results are very similar when the HER2/CEN 17 ratio cutoff is set at 2. In this situation, the number of patients eligible for Trastuzumab is higher for SISH (n=6) and qPCR (n=4) and identical for CISH.

### Concordance between FISH and alternative techniques

The results of IHC, FISH and alternative techniques are presented in Tables 1 and 2, expressed in terms of the HER2/CEN17 ratio in 3 categories and the *HER2 copy* number, respectively. Concordances and predictive values are presented in Tables 3 and 4.

**Table 3:** Predictive value of each alternative technique compared with FISH expressed as HER2/CEN17 ratio in 3 categories in the overall population (n=766) and in the IHC 2+ subpopulation

**Techniques**	**Population**	**Concordance (95%CI)**	**Sensitivity (95%CI)**	**Specificity (95%CI)**	**Positive predictive value (95%CI)**	**Negative predictive value (95%CI)**
IHC	All (N=766)	96% [94-97]	91% [86-94]	98% [97-99]	95% [91-98]	96% [94-98]
SISH	All (N=498)	97% [96-99]	99% [95-100]	97% [94-98]	93% [88-96]	99% [98-100]
	IHC 2+ (N=54)	89% [77-96]	80% [44-97]	91% [78-97]	67% [35-90]	95% [84-99]
CISH	All (N=108)	98% [93-100]	100% [89-100]	97% [91-100]	94% [80-99]	100% [95-100]
	IHC 2+ (N=18)	100% [81-100]	100% [16-100]	100% [79-100]	100% [16-100]	100% [79-100]
qPCR	All (N=699)	95% [93-96]	89% [83-93]	97% [95-98]	92% [88-96]	95% [93-97]
	IHC 2+ (N=86)	93% [85-97]	73% [45-92]	97% [90-100]	85% [55-98]	95% [87-98]

CI: confidence interval.

**Table 4:** **Predictive value of each alternative technique compared with FISH expressed as *HER2 *copy number (cutoff set at 6 *HER2 *copies) in the overall population (n=840) and in the IHC 2+ subpopulation**

**Techniques**	**Population**	**Concordance (95%CI)**	**Sensitivity (95%CI)**	**Specificity (95%CI)**	**Positive predictive value (95%CI)**	**Negative predictive value (95%CI)**
IHC	All (N=840)	95% [94-97]	89% [84-92]	98% [97-99]	95% [92-98]	95% [93-97]
SISH	All (N=587)	98% [96-99]	95% [91-98]	99% [98-100]	98% [94-99]	98% [96-99]
	IHC 2+ (N=60)	90% [81-96]	72% [47-90]	96% [87-100]	87% [60-98]	91% [81-97]
CISH	All (N=204)	75% [69-81]	99% [92-100]	64% [56-72]	57% [48-67]	99% [94-100]
	IHC 2+ (N=27)	62% [42-79]	100% [40-100]	56% [35-76]	27% [8-55]	100% [77-100]
qPCR	All (N=773)	93% [91-94]	80% [75-85]	98% [96-99]	94% [90-97]	92% [90-94]
	IHC 2+ (N=86)	86% [78-92]	45% [24-68]	97% [91-100]	83% [52-98]	86% [77-93]

CI: confidence interval.

Each center was required to perform IHC, FISH, SISH or CISH and qPCR for each case. For various reasons, this objective was not achieved and some participants were unable to perform all methods.

**Figure 1:**
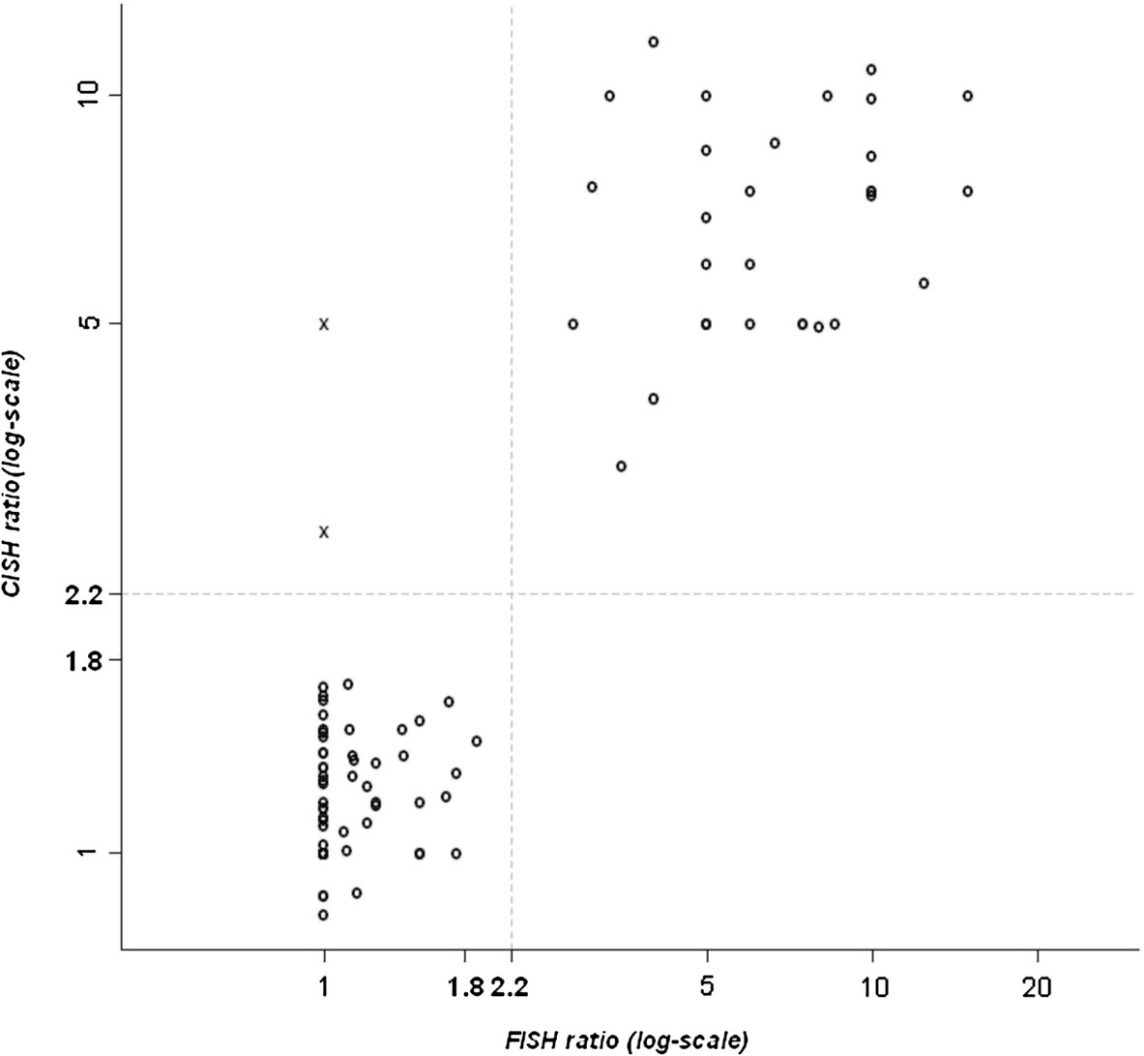
Correlation between FISH and CISH in terms of HER2/CEN17 ratio mentioning the three categories cutoff.

**Figure 2:**
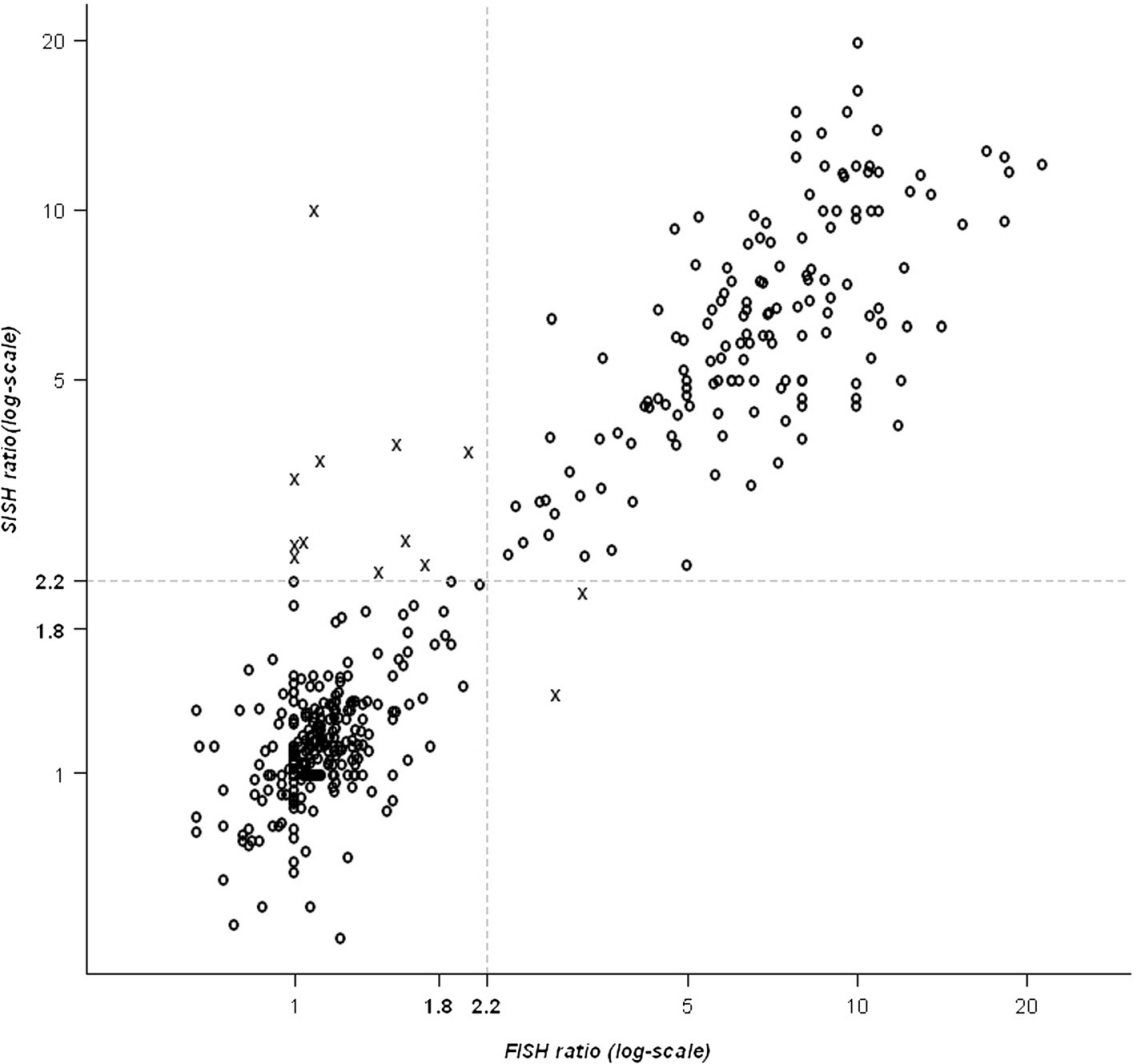
Correlation between FISH and SISH in terms of HER2/CEN17 ratio mentioning the three categories cutoff.

Among the 498 cases evaluable by SISH with FISH expressed as a ratio, 156 had an HER2/CEN17 ratio greater than 2.2 (Table 1) and a global concordance of 97% with FISH (Table 3). The Figure 1 shows the correlation between FISH and CISH in terms of HER2/CEN17 ratio expressed in 3 categories. Among the 587 cases evaluable by SISH with FISH expressed as *HER2* copy number, 170 cases had an *HER2* copy number greater than 6 (Table 2) with a global concordance of 98% with FISH (Table 4). The Figure 2 shows the correlation between FISH and SISH in terms of HER2/CEN17 ratio expressed in 3 categories.

Among the 108 cases analyzed by CISH with FISH expressed as a ratio, 33 had an HER2/CEN17 ratio greater than 2.2 (Table 1) and a global concordance 98% with FISH (Table 3). On the 115 of cases who had an *HER2 copy* number greater than 6 (Table 2) we observed only 75% concordance between the two methods (Table 4).

**Figure 3:**
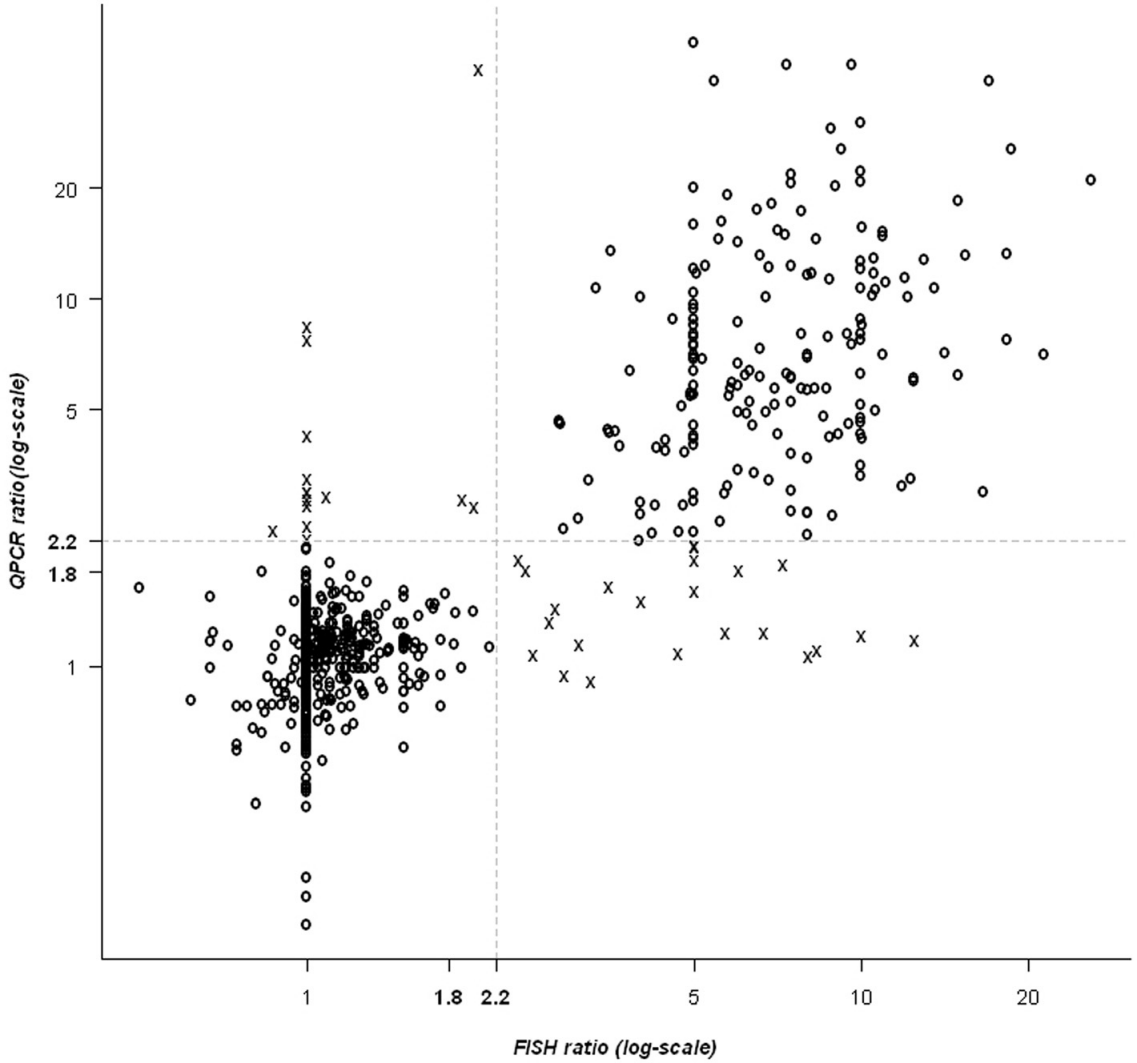
Correlation between FISH and qPCR in terms of HER2/CEN17 ratio mentioning the three categories cutoff.

Of the 699 cases analyzed by qPCR with FISH expressed as a ratio, 195 cases had an HER2/CEN17 ratio greater than 2.2 (Table 1) and a global concordance of 95% with FISH (Table 3). The Figure 3 shows the correlation between FISH and qPCR in terms of HER2/CEN17 ratio expressed in 3 categories.

Among the 773 cases analyzed by qPCR with FISH expressed as *HER2* copy number, 197 had an *HER2* copy number greater than 6 (Table 2) corresponding to 93% of global concordance with FISH (Table 4).

### Predictive value of each alternative technique

The sensitivity of qPCR appeared to be slightly lower than that of CISH and SISH. However, a higher sensitivity was observed when FISH was expressed as the HER2/CEN17 ratio in 3 categories (Table 3) than when it was expressed as *HER2* copy number (Table 4). A very high specificity (97%) was observed for 3 alternative techniques when FISH was expressed as the HER2/CEN17 ratio. When FISH was expressed as *HER2* copy number, SISH and qPCR were associated with very high specificities (99% and 98% respectively), while the specificity of CISH was only 64%.

When the HER2/CEN17 ratio cutoff was set at 2, the level of predictive value is quite similar (Additional file 1: Table S4).

### Predictive value of alternative techniques in the subpopulation of 2+ cases

A marked heterogeneity was observed between the 15 centers in terms of the proportion of amplified cases (FISH HER2/CEN17 ratio greater than 2.2) among the 2+ cases, varying from 0% in three centers to less than 25% in nine centers and more than 25% in the other three centers. The results, especially the CISH results, must be interpreted cautiously in view of the small number of 2+ cases analyzed by an alternative technique (86 for qPCR, 54 for SISH and only 18 for CISH). Indeed, CISH appeared to have very good predictive values for 2+ cases, but this could not be formally demonstrated due to the small number of cases.

When FISH was expressed as a ratio in 3 categories (Table 3), the highest concordance was observed for CISH (100%) followed by qPCR (93%) and SISH (89%). When FISH was expressed as copy number (Table 4), the highest concordance was observed for SISH (90%) followed by qPCR (86%) and CISH (62%).

Except for CISH, lower sensitivity was observed when FISH was expressed as *HER2* copy number rather than HER2/CEN17 ratio (72% for SISH and 45% for qPCR). When FISH was expressed as HER2/CEN17 ratio, the highest specificity was observed for SISH and very similar results were observed for qPCR (97%) and for CISH (91%). When FISH was expressed as *HER2* copy number, high specificities were observed for SISH and qPCR, while CISH specificity was only 56%.

When the HER2/CEN17 ratio cutoff was set at 2, the level of predictive value is quite similar; higher specificity level was observed for qPCR (Additional file 1: Table S4).

## Discussion

We have previously demonstrated the accuracy of *HER2* determination on core biopsies with respect to surgical resection by using alternative techniques to FISH such as CISH and SISH [17]. The present multicenter study, performed on consecutive cases from 15 French institutions, is the largest series performed on paraffin-embedded diagnostic core biopsies, demonstrating correlations between IHC, FISH and additional alternative methods i.e. CISH or SISH and qPCR. Few analyses have been done on needle core biopsies exclusively and in a so large multicenter manner. Analysis of core biopsies represents real clinical practice for patients receiving neoadjuvant therapy. The global concordance of IHC with FISH in these cases was excellent and comparable to that reported in previous studies mainly performed on surgical specimens [9,31-33]. Each of the 15 participating institutions had to use the alternative “in situ” technique used routinely in their respective laboratory, which explains why only 204 cases of this series were analyzed by CISH. Following completion of this study, CISH was widely replaced by SISH, which is more rapid, more reproducible and more easily automated [31]. However in this study, the concordance between CISH and FISH was excellent and similar to that reported in previous studies [34-37]. SISH gave also an excellent concordance with FISH in this series, i.e. comparable to that previously reported [18-21], for example Papouchado’s study based on 298 surgical specimens who reported a concordance of 92.1% with a high level of reproducibility (96.6%) between ten pathologists [18].

The correlation between FISH and qPCR was also excellent. Despite the heterogenous variable expertise of the various participants at the beginning of the study, the preliminary training steps, the common protocol and common controls and reagents resulted in an excellent yield, as only seven cases were not interpretable and only three cases had to be repeated in a second series of slides (not shown). Molecular analysis was performed on paraffin-embedded core biopsies in contrast with most published studies, which were generally performed on frozen surgical material [22-25]. Some studies were performed on paraffin sections but were based on a small number of cases and used the HER-2/neu Quantification Kit™ developed by Roche for a LightCycler platform [26-28]. This kit has now been withdrawn from the market, as it was shown to be not optimal to detect chromosome 17 polysomy [29]. With a concordance of 95% with FISH, the results of the present study are comparable to those of previous studies [23-29]. The qPCR assay designed for this study was performed with five probes located on chromosome 17 (arm 17q) to distinguish between chromosome 17 polysomy and *HER2* focal amplification. Overall, the correlation with FISH was better in the overall population and in the 2+ cases when the results were expressed as the HER2/CEN17 ratio, suggesting that this is a suitable approach. The potential disadvantage of qPCR is that it cannot avoid dilution artefacts inherent to DNA extraction in heterogeneous tumor specimens. Macrodissection is now used in the routine detection of *KRAS, EGFR, BRAF*… mutations and its systematic use in this study could have further improved the performances of qPCR in cases with low cellularity. However, the high level of cellularity (median value of 60%) and the low percentage of in situ component observed in this series should be stressed. These results on paraffin sections are very encouraging for routine clinical practice, as, when studying paraffin-embedded material, DNA material is easier to use than RNA, which is more sensitive to fixation conditions and degradation, resulting in a potential risk of *HER2* misclassification [38]. At last, the high throughput capacity of qPCR and its attractive cost price are worth noting.

We observed very similar results using a HER2/CEN17 ratio expressed in 3 categories or in 2 categories with a cutoff set at 2, as it has been recently suggested.

These alternative techniques to FISH could be used in routine practice as a primary test or to more reliably evaluate ambiguous 2+ cases, particularly in the neoadjuvant setting. The frequency of *HER2* amplification demonstrated by FISH in 2+ cases is consistent with the results published in the literature [31,39] with 16.8% of cases presenting an HER2/CEN17 ratio greater than 2.2 and 21.1% of cases presented an *HER2* copy number greater than 6. In general, the 2+ cases present low levels of amplification and low copy numbers depending on the percentage of true complete membrane staining [40]. It has been demonstrated that this category may present high rates of polysomy, which would explain why a high copy number can be associated with an HER2/CEN17 less than 2.2 [41], consequently improving the positive predictive value with the true *HER2* copy number. An excellent concordance with FISH was observed for both SISH and qPCR, the sensitivity was lower than in the overall population, but nevertheless associated with a better positive value for qPCR when FISH is expressed as the HER/CEN17 ratio. These results suggest that the use of several probes to estimate polysomy provides a better correlation with FISH than when only the *HER2* copy number is used. Equivalent positive predictive values were obtained when these two techniques were based on *HER2* copy number.

Another important point is the fact that 2+ cases are considered to be the most heterogeneous category, explaining the discordance between core biopsies and surgical specimens [42] and the technique cannot be repeated on surgical specimens in the neoadjuvant setting. These discrepancies are observed more frequently around the cut-off used to define positivity [40].

The marked variation between centers in terms of the level of amplification in 2+ cases suggests that the IHC technique must be more closely standardized for these cases. According to the guidelines, 2+ amplified cases must be included in external controls for IHC [6,7,35], but these cases are rare and present amplification in less than 25% of cases, for example 14% in the Nottingham series [42]. The 2+ cases correspond to the cases with the most marked genetic heterogeneity [43] with a lower level of amplification compared to 3+ cases [44]. This amplification is related to the percentage of positive membrane staining cells [42]. Under these conditions, needle core biopsies are the least appropriate specimens for 2+ cases. All of these borderline and heterogeneous situations therefore required repeated analysis or the use of alternative techniques.

Medico-economic aspects must also be taken into account in the choice of method. The medico-economic study is ongoing in the same patient series, but the results are not reported here.

## Conclusions

This multicenter study shows that SISH, CISH and qPCR alternative techniques to evaluate *HER2* amplification status are easy to use and provide encouraging results. In ambiguous cases scored 2+ by IHC, the heterogeneity, the small proportion of amplified cases, and the lower *HER2* copy number must be taken into account in the neoadjuvant setting to assess *HER2* status on core biopsies. In this case, the use of various alternative techniques such as SISH and qPCR could be a reliable approach.

The qPCR protocol used in the 15 participating institutions was found to be an acceptable alternative to FISH to detect *HER2* amplification in paraffin-embedded material.

## Methods

### Patient selection

This study is a non-interventional study and no written consent was needed. In agreement with the French legislation, the protocol was approved by the Comité Consultatif sur le Traitement de l’Information en matière de Recherche dans le domaine de la Santé (CCTIRS) and declared to the Commission Nationale de l’Informatique et des Libertés (CNIL).

The required number of patients was estimated according to the expected FISH positivity of each IHC level according to ASCO/CAP 2007 [3]: 840 cases were necessary: 0=317 (38%), 1+= 183 (22%), 2+= 109 (13%), 3+ =231 (27%). Each of the 15 centers complied with this proportion by recruiting a mean of 56 cases.

Tumor cell percentage and presence of an in situ component were assessed on Hematein/Eosin stained sections.

A representative block of fixed paraffin-embedded tumor tissue from each patient was selected and used to prepare sections for IHC and FISH/SISH/CISH. Four additional 10-μm sections were taken from the same block for DNA extraction and qPCR.

### a) IHC and in situ hybridization

All 15 centers participate in the French national annual quality control (AFAQAP) and are members of the GEFPICS group [4,9]. According to French guidelines, the choice of method (IHC vs ISH, brand of antibodies or ISH kits) is left to the pathologist’s discretion. The list of the antibodies used by the participants is given in Additional file 1: Table S1. As different fixatives are used in routine practice, a training step was conducted before initiation of this study using two types of tissue micro-arrays (TMA) representative of the fixative used in each center. A 0.6 mm diameter needle was used for the TMA. The first TMA was performed with alcohol formalin (AF) used by 41% of the participants, the second TMA was performed with formalin (F) used by the remaining 59% of participants. Each TMA included 13 cases 6/3+, 2/2+ 3/1+ and 2/0 and 3 control cell lines with various *HER2* amplification levels (T47D, MCF7, BT474). The amplification level of each case was evaluated by FISH by two of the authors (JJ/FPL).

Each participant was required to validate IHC and CISH or SISH and FISH in situ techniques on the TMA depending on the fixative used in the center (AF or F). The good concordance obtained (92%) allowed initiation of the study. The cases included in these TMA were also used for qPCR training.

The 2007 ASCO/CAP [3,30] guidelines were used to define IHC categories: negative = no membrane staining, 1+ = faint or barely perceptible membrane staining, 2+ = 10-30% of strong complete membrane staining or >10% tumor cells with moderate complete membrane staining, 3+ = more than 30% strong complete membrane staining.

A minimum of 20 tumor nuclei are required for in situ hybridization on core biopsy. FISH was performed with a dual probe kit (HER2 and CEN17) *HER2* FISH pharmDx™ (Dako France SAS, Trappes, France) or Vysis Path Vysion (Abbott France SAS, Rungis, France). A mono-probe kit INFORM (Ventana Medical Systems SA, Illkirch, France) was used in one center. SISH was performed in 10 centers using the Ventana Kit on Benchmark XT(Roche Diagnostic, Meylan, France) (http://www.ventana.com). CISH was performed in 5 centers according to the DAKO or Cytovision kits (Leica/Cytovision, Nanterre, France). The following cut-offs were used according to the 2007 ASCO/CAP [3,30] guidelines: amplified (R >2.2) borderline amplification (1.8-2.2) and non amplified (<1.8). A second analysis was performed with the cutoff set at 2: amplified (R>=2) and non amplified (R<2).

### b) qPCR

qPCR was coordinated by two of the authors (FS, IB) at the Institut Curie - Hôpital René Huguenin (IC-HRH). All 15 participants used the same DNA extraction procedure (QIAamp kit, Qiagen). The qPCR method was performed in 14 centers; samples from one center were blindly analyzed in the coordinator’s lab without knowledge of the IHC or FISH status. One centre participated to the preliminary steps of the study but did not perform the prospective study.

At the beginning of the project, participants had different levels of expertise in PCR assays. Three rounds of tests were organized before initiating the prospective study in order to test and standardize practices and to define the most appropriate primer pairs for evaluation of *HER2* amplification, chromosome 17 polysomy and diploidy controls. The reference genes, located on the same chromosome as *HER2*, provide a control for DNA quality and loading and are also used as an internal control gene to evaluate chromosome 17 polysomy. The choice of the probes used to detect polysomy was focused on the 17q arm.

A total of 22 primer pairs were tested in three preliminary tests, 7 for *HER2*, 8 for evaluation of chromosome 17 polysomy and 7 for diploidy control. Primer pairs were chosen on the basis of CGH array studies performed at the IC/HRH, published data and previous experience of *HER2* amplification on frozen breast tumors [22]. The initial tool was modified during the course of the 3 preliminary tests. The following material was used to test the evolving tool: three consecutive series of 15 breast cancer tumors provided by the participants (secondarily anonymized and redistributed by the coordinator) with known *HER2* status; appropriate controls (normal lymph nodes, cell lines) and cases with various DNA qualities to test the robustness of the assay.

The coordinators proposed a final common protocol to the participants for the prospective study, including 10 sets of primer pairs: 2 for *HER2* (exons 8 and 26), 5 for chromosome 17 polysomy detection: *TAOK1 at* 17q11.2 (sub-centromeric region), *UTP6* at 17q11.2 (sub-centromic region), *MRM1* at 17q12, *MKS1* at 17q22 and *SSTR2 at* 17q24, and 3 for diploidy control: *TSN* at 2q14, *LAP3* at 4p15 and *ADAMTS16* at 5p15 (Additional file 1: Table S2).

Participants received detailed instructions on the technique and vials containing the oligonucleotides for the 10 genes and for the prospective cases to be tested, together with DNA extracted from normal lymph nodes and SKBR-3 breast cancer cell lines, as controls for non-amplified and amplified HER-2 gene copy, respectively. PCR analyses were performed in duplicate in a 10 μL reaction volume. A quantity of 200 ng DNA was necessary for each patient for duplicate analysis of the ten genes (10 ng/PCR reaction). Each participant used his/her own quantitative PCR platform. Considering the threshold of each device, most participants used Applied Biosystems material with the same threshold set at 0.20 for the Appled 7900 for example. For the other devices, threshold was adapted during the preliminary steps of the study. Participants also received Excel sheets with pre-calculated areas from individual Ct values obtained for each gene. In order to be comparable with FISH and SISH/CISH, qPCR data were expressed as the median copy number of the 5 genes used for “chromosome 17 control” and assessment of polysomy, and the median HER2 copy number. Finally, these two numbers were used to calculate the HER2/CEN17 ratio. Cycle threshold (Ct) values above 35 were excluded. Cut-offs used for *HER2* amplification status by qPCR were similar to those used in in situ methods.

A database was created with the following parameters provided by the participants:

Hematein-Eosin staining: histological type, percentage of tumor cellularity, proportion of in situ component, grade, core size, fixative, fixation time.

Immunohistochemistry: antibody and dilution, type of pre-treatment, ASCO/CAP 2007 score with the percentage of positive cells and intensity [3].

FISH: 1) name of the kit used; 2) number of nuclei analyzed; 3) absolute and mean *HER2* copy number; 4) absolute and mean Chromosome 17 number; 5) HER2/CEN17 ratio and final results with the cut-off values according to ASCO/CAP 2007 guidelines:; 5) the number of times the technique was repeated, when applicable.

CISH and SISH were analyzed according to the same plan.

qPCR: 1) Quality of the DNA 260/280 absorbance ratio (evaluated by Nanodrop™); 2) *HER2* copy number; 3) reference gene copy number; 4) final ratio for *HER2* status using the same cut-off values as for FISH; 5) the number of times the technique was repeated, when applicable.

### Statistical analysis

The sample size was calculated to ensure a lower boundary of the 95% confidence interval of sensitivity and specificity to be upper than 80%. This calculation was based on the CI95’s formula for a proportion.

Data were summarized by frequency and percentage for categorical variables and the median and range were computed for continuous variables.

The predictive capabilities of each experimental technique were described by concordance percentage, sensitivity, specificity, and positive and negative predictive values, considering FISH as the reference technique.

A sample size of 850 cases was required to demonstrate sensitivity and specificity greater than 80%, on the basis of the lower boundary of the 95% confidence interval of a proportion. A total of 840 evaluable cases were finally included.

Statistical analysis was performed using R.2.15.0 software.

## Abbreviations

AF: Alcohol formol; AFAQAP: Association Française d’Assurance Qualité en Anatomie et cytology Pathologiques; ASCO/CAP: American Society of Clinical Oncology/College of American Pathologists; CEN17: Centromere chromosome 17; CISH: Chromogenic in situ hybridization; CNIL: Commission Nationale de l’Informatique et des Libertés; CCTIRS: Comité Consultatif sur le Traitement de l’Information en matière de Recherche dans le domaine de la Santé; F: Formol; FISH: Fluorescent in situ hybridization; GEFPICS: Groupe d'Etude des Facteurs Pronostiques IHC dans le Cancer du Sein; IHC: Immunohistochemistry; ISH: In situ hybridization; qPCR: Quantitative real-time polymerase chain reaction; SISH: Silver-enhanced in situ hybridization; TMA: Tissue microarray.

## Competing interests

The authors declare that they have no competing interests.

## Authors’ contributions

JJ conceived of the study and its design, coordinated and drafted the manuscript. FS conceived of the study and its design, coordinated the molecular studies and drafted the manuscript. IB coordinated the molecular studies and helped to draft the manuscript. FPL conceived of the study and participated in its design and helped to draft the manuscript. BE participated in the design of the study and performed the statistical analysis. SV participated to the coordination of the qPCR assays. AV contributed to FISH interpretation in the preliminary steps. JJ, MA, LA, PB, CB-F, FB, MPC, JMG, DL, GMG, MCM, YMR, ML-T, AV-S, LX, FPL supervised or carried out the IHC, FISH and SISH or CISH. MJM, SL, JL-C, SK, PJL, ML-J, IH, LL, FR, AS, PDC, GP supervised or carried out the qPCR assays. All authors read and approved the final manuscript.

## Pre-publication history

The pre-publication history for this paper can be accessed here:

http://www.biomedcentral.com/1471-2407/13/351/prepub

## Supplementary Material

Additional file 1: Table S1 List of the antibodies used for immunohistochemistry according to the french and AFAQAP guidelines. **Table S2.** List of primers used for the qPCR method. **Table S3.** Distribution of the 766 cases analyzed by double probe FISH expressed as HER2/CEN17 ratio with a cutoff set at 2 with respect to the CISH, SISH, and QPCR alternative techniques. **Table S4.** Predictive value of each alternative technique compared with FISH expressed as HER2/CEN17 ratio with a cutoff set at 2 in the overall population (n=766) and in the IHC 2+ subpopulation. Click here for file
