# Supplementary material for: SISH/CISH or qPCR as alternative techniques to FISH for determination of HER2 amplification status on breast tumors core needle biopsies: a multicenter experience based on 840 cases
Source: BMC Cancer. 2013 Jul 22;13:351. doi: 10.1186/1471-2407-13-351 (PMC3729815; doi:10.1186/1471-2407-13-351)
Supplement: Additional file 1: Table S1 — List of the antibodies used for immunohistochemistry according to the french and AFAQAP guidelines. Table S2. List of primers used for the qPCR method. Table S3. Distribution of the 766 cases analyzed by double probe FISH expressed as HER2/CEN17 ratio with a cutoff set at 2 with respect to the CISH, SISH, and QPCR alternative techniques. Table S4. Predictive value of each alternative technique compared with FISH expressed as HER2/CEN17 ratio with a cutoff set at 2 in the overall population (n=766) and in the IHC 2+ subpopulation. [file 1471-2407-13-351-S1.docx]

Supplementary Table 1: List of the antibodies used for immunohistochemistry according to the french and AFAQAP guidelines

| Manufacturer | **Clone** | **Number of centre** | **Number of Patients** |
| --- | --- | --- | --- |
| **DAKO** | Polyclonal AO485 | 7 | 420 (50%) |
| **DAKO** | Polyclonal AO485  HERCEPTEST KIT | 1 | 60 (7%) |
| **NOVOCASTRA** | NCLCB11 | 2 | 118 (14%) |
| **VENTANA** | 4B5 | 5 | 242 (29%) |
| **Total** |  | 15 | 840 |

Dako France SAS, Trappes, France ,Novocastra /Menarini France , Ventana Medical Systems SA, Illkirch, France

| **Gene** | **Localisation** | **Sequence** | **Size (bp)** |
| --- | --- | --- | --- |
| **ERBB2** | **17q21.1** | U : 5'- ACG GAC GTG GGA TCC TGC A -3' | 84 |
|  |  | L : 5'- CTT CTC ACA CCG CTG TGT TCC AT -3' |  |
|  |  | U : 5'- ACA TGA CCC CAG CCC TCT ACA -3' | 80 |
|  |  | L : 5'- GGG CAA CGT AGC CAT CAG TCT -3' |  |
| **TAOK1** | **17q11.2** | U : 5'- CAG GCC AGG TGA AAC TTG CTG A -3' | 75 |
|  |  | L : 5'- ACG GCG TTC CCA CAA AGG AA -3' |  |
| **UTP6** | **17q11.2** | U : 5'-CCT TGG TAG ACC TGA GAA CTG TGG A-3' | 88 |
|  |  | L : 5'-TTT AGC TAC AAA TGC CTC TGC TGA CT-3' |  |
| **MRM1** | **17q12** | U : 5'- TTT GGG GCT GTG CTG CGT T -3' | 68 |
|  |  | L : 5' - CTG TTT CTC CGG CTG GTG ATG A - 3' |  |
| **MKS1** | **17q22** | U : 5'- CCT GTG TAC TCT GAA GGT GGA TAG CA -3' | 59 |
|  |  | L : 5'- CCG TGA AGT CAG GCT TTA CTG TGA T -3' |  |
| **SSTR2** | **17q24** | U : 5'- GGT CAA GGT GAG CGG CAC AGA T - 3' | 63 |
|  |  | L : 5' - AGC CGG GAT TTG TCC TGC TTA CT - 3' |  |
| **TSN** | **2q14** | U : 5'- CAG CGT GAC TGC TGG AGA CTA CT -3' | 70 |
|  |  | L : 5'- ACC GGA ATC CAG CTC ATT GAT -3' |  |
| **LAP3** | **4p15** | U : 5' - TGA TGC TGA GGG GAG GCT CAT A -3' | 62 |
|  |  | L : 5'- TCG GGT TAA ACG TGT GTG CGT A -3' |  |
| **ADAMTS16** | **5p15** | U : 5'- TGG TCT CTG CCT ACG AGG TTG A -3' | 69 |
|  |  | L : 5'- GCC GCT GAT GGT GCA TGA T -3' |  |

Supplementary table 2: List of primers used for the qPCR method

Supplementary Table3: Distributionof the 766cases analyzedby double probe FISH expressed as HER2/CEN17 ratio with a cutoff set at 2 with respect to the CISH, SISH, and QPCR alternative techniques.

| **Techniques** | **Class** | **Number of cases** | **FISH Ratio <2**  **N=539 (%)** | **FISH Ratio >=2**  **N=227 (%)** |
| --- | --- | --- | --- | --- |
| IHC | 0 | 287 | 284 (99%) | 3 (1%) |
|  | 1+ | 172 | 169 (98%) | 3 (2%) |
|  | 2+ | 95 | 77 (81%) | 18 (19%) |
|  | 3+ | 212 | 9 (4%) | 203 (96%) |
| SISH | <2 | 336 | 334 (99%) | 2 (1%) |
|  | >=2 | 162 | 14 (9%) | 148 (91%) |
|  | ND or NA | 268 | 191 | 77 |
| CISH | <2 | 75 | 75 (100%) | 0 (0%) |
|  | >=2 | 33 | 2 (6%) | 31 (94%) |
|  | ND or NA | 658 | 462 | 196 |
| QPCR | <2 | 500 | 477 (95%) | 23 (5%) |
|  | >=2 | 199 | 15 (8%) | 184 (92%) |
|  | ND or NA | 67 | 47 | 20 |

Supplementary Table 4: Predictive value of each alternative technique compared with FISH expressed as HER2/CEN17 ratio with a cutoff set at 2 in the overall population ( n=766) and in the IHC 2+ subpopulation

| Techniques | Population | Concordance | Sensitivity | Specificity | Positive Predictive Value | Positive Predictive  Value |
| --- | --- | --- | --- | --- | --- | --- |
| IHC | All (N=766) | 96%  [94-97] | 91%  [86-94] | 98%  [97-99] | 95%  [91-98] | 96%  [94-98] |
| SISH | All (N=498) | 97%  [95-98] | 99%  [96-100] | 95%  [93-97] | 90%  [84-94] | 100%  [98-100] |
|  | IHC 2+ (N=54) | 87%  [75-95] | 90%  [55-100] | 86%  [73-95] | 60%  [32-84] | 97%  [87-100] |
| CISH | All (N=108) | 98%  [93-100] | 100%  [89-100] | 97%  [91-100] | 94%  [80-99] | 100%  [95-100] |
|  | IHC 2+ (N=18) | 100%  [81-100] | 100%  [16-100] | 100%  [79-100] | 100%  [16-100] | 100%  [79-100] |
| Q-PCR | All (N=699) | 95%  [93-96] | 90%  [85-93] | 97%  [95-98] | 91%  [87-95] | 96%  [94-97] |
|  | IHC 2+ (N=86) | 93%  [85-97] | 73%  [45-92] | 97%  [90-100] | 85%  [55-98] | 95%  [87-98] |
